# BRG1 orchestrates diabetic corneal neuropathy via PI3K/AKT-mediated glycolytic reprogramming

**DOI:** 10.1186/s40662-026-00474-4

**Published:** 2026-02-01

**Authors:** Yuyang Deng, Wenqu Chen, Danling Liao, Jianzhang Hu

**Affiliations:** Department of Ophthalmology, Fujian Medical University Union Hospital, 29 Xinquan Road, Fuzhou, 350005 China

**Keywords:** Diabetic corneal neuropathy, Glycolysis, Brahma-related gene 1, PI3K/AKT signaling pathway, Corneal nerve regeneration

## Abstract

**Background:**

Mounting evidence indicates metabolic dysregulation in diabetic corneal neuropathy (DCN). This study elucidates how the chromatin remodeler Brahma-related gene 1 (BRG1) orchestrates glycolytic reprogramming to drive neurodegeneration and epithelial repair defects in DCN.

**Methods:**

Type 1 diabetic mice were established via streptozotocin (STZ) injection. Glycolysis was inhibited using 2-deoxy-D-glucose (2-DG) to assess its role in DCN pathogenesis. BRG1 expression was modulated by subconjunctival plasmid delivery (overexpression/knockdown). Pathway screening identified BRG1 downstream effectors, and phosphatidylinositol 3-kinase/protein kinase B (PI3K/AKT) inhibition (LY294002) confirmed regulatory hierarchy. Glycolytic flux was evaluated via Western blotting and immunofluorescence; corneal nerve integrity and epithelial healing were assessed by βIII-tubulin staining and sodium fluorescein assay.

**Results:**

Hyperglycemia upregulated BRG1 and glycolytic enzymes in diabetic corneal nerves. BRG1 overexpression exacerbated epithelial repair delay and neurodegeneration, while knockdown partially reversed damage. BRG1 overexpression activated PI3K/AKT transcription, and PI3K/AKT inhibition did not alter BRG1 levels but rescued BRG1-induced pathologies.

**Conclusions:**

Glycolytic reprogramming is a critical driver of DCN progression. BRG1 activates PI3K/AKT signaling to enhance glycolytic flux, thereby regulating DCN pathogenesis. Targeting this axis may offer novel therapeutic strategies.

**Supplementary Information:**

The online version contains supplementary material available at 10.1186/s40662-026-00474-4.

## Background

Diabetic keratopathy, as one of the ocular complications of diabetes, is specifically characterized by persistent epithelial defects, superficial punctate keratitis, delayed corneal healing, and other severe refractory symptoms that markedly impair visual function and quality of life [1]. The cornea is innervated by the trigeminal nerve and represents the most densely innervated structure in the human body. These nerve fibers, which originate from the trigeminal nerve and form an intricate plexus within the cornea, play a critical role in corneal sensation (including pain, touch, and thermal perception) and the blink reflex [2]. Furthermore, through the release of various neurotransmitters and neuropeptides—such as epidermal growth factor (EGF), neuropeptides, and calcitonin gene-related peptide (CGRP)—they work in concert with epithelial cells to maintain the delicate biochemical balance essential for corneal epithelial regeneration [3, 4]. Chronic hyperglycemia induces persistent functional impairment of corneal nerves and disrupts neurotrophic support systems, thereby increasing epithelial fragility and diminishing repair capacity—ultimately establishing a pathological feedback loop that constitutes the central focus of this investigation [5, 6]. Current research on diabetic corneal neuropathy (DCN) primarily encompasses multiple pathological pathways. Systematically elucidating the molecular basis underlying DCN and identifying novel therapeutic targets represent pressing priorities in the field.

Glycolysis, a ten-step enzymatic process, converts glucose into pyruvate under normoxic conditions with a net production of two adenosine triphosphate (ATP) molecules [7]. As the central pathway of glucose metabolism, glycolysis plays a pivotal role in maintaining corneal energy homeostasis. However, in hyperglycemic environments, cells may shift from oxidative phosphorylation to glycolysis via multiple pathological mechanisms, leading to energy crisis and lactate accumulation [8–11]. Glycolytic flux is regulated by diverse signaling pathways, including the phosphatidylinositol 3-kinase/protein kinase B (PI3K/AKT) pathway [12], adenosine monophosphate-activated protein kinase (AMPK) pathway [13], and cyclic GMP-AMP synthase/stimulator of interferon genes (cGAS/STING) signaling [14]. Notably, the PI3K/AKT signaling pathway, a critical hub for intracellular proliferation and metabolic regulation, promotes glucose uptake and glycolysis by modulating glucose transporter 1 (GLUT1) and key glycolytic enzymes such as hexokinase 2 (HK2) and lactate dehydrogenase A (LDHA), thereby sustaining cellular energy supply [15–17]. Although the functional interplay between PI3K/AKT signaling and metabolic reprogramming is well-documented, its crosstalk with epigenetic regulators in DCN remains unexplored.

Brahma-related gene 1 (BRG1), the catalytic ATPase subunit of the switch independent/sucrose non-fermentable (SWI/SNF) complex, serves as a core component of multiple multiprotein complexes [18, 19]. Studies demonstrate that BRG1 deficiency induces metabolic reprogramming in cancer cells, characterized by reduced glucose uptake and suppressed glycolysis [20]. BRG1 enhances the transcriptional activity of glycolytic gene promoters (e.g., HK2, PKM2, phosphofructokinase-1 [PFK-1]) and modulates glycolysis by regulating key metabolites such as glucose-6-phosphate, fructose-6-phosphate, and inositol [21, 22]. BRG1 additionally functions as a histone modification reader, directly governing gene expression through modulation of histone marks—including histone H3 lysine 27 trimethylation (H3K27me3), histone H3 lysine 9 trimethylation (H3K9me3), histone H3 lysine 27 acetylation (H3K27ac), and histone H3 lysine 14 acetylation (H3K14ac)—thereby contributing to glycolytic control [22, 23]. These findings establish BRG1 as a metabolic hub in glycolytic regulation across various diseases. However, its role in glycolytic reprogramming within DCN remains completely unexplored.

In this study, we investigate the expression and regulation of glycolysis in DCN through targeted modulation of BRG1 and glycolytic pathways, with a primary focus on elucidating the potential role of BRG1 in this context. Our objectives are to delineate the function of BRG1 in DCN and its association with glycolysis, ultimately aiming to identify novel therapeutic targets and advance the development of treatment strategies for diabetic corneal complications.

## Methods

### Experimental animals

Male C57BL/6 mice (6–8 weeks old, weighing 20–25 g) were obtained from SPF Biotechnology Co., Ltd. (Beijing, China). All mice used in the study were housed and cared for at the Animal Experiment Center of Fujian Medical University (FJMU) by professional staff. The treatment of the animals was reviewed and approved by the Institutional Animal Care and Use Committee (IACUC) of Fujian Medical University (IACUC FJMU 2021-0454) and strictly adhered to the ARVO Statement for the Use of Animals in Ophthalmic and Vision Research. A type 1 diabetes mouse model was established via intraperitoneal injection of streptozotocin (STZ; Sigma-Aldrich, USA) dissolved in citrate buffer (50 mg/kg) for 5 consecutive days. Control mice received equivalent volumes of citrate-citric acid buffer via intraperitoneal injection. Body weight and tail vein blood glucose levels were measured prior to the initial injection. Blood glucose levels were subsequently monitored every 4 weeks until week 16, with the average value calculated from three consecutive measurements at each time point. Mice exhibiting an average blood glucose measurement exceeding 350 mg/dL (16.7 mmol/L) were considered to have successful model induction. Blood glucose was rechecked prior to each subsequent experimental use of the mice.

### Corneal sensitivity measurement

Corneal sensitivity was measured in unanesthetized mice using a Cochet-Bonnet aesthesiometer (Luneau Ophtalmologie, France) bilaterally. A blink reflex was defined as a positive response. Testing started with the nylon filament at its maximum length of 6 cm. If no positive response occurred, the filament was shortened by 0.5 cm until a blink reflex appeared. The filament length at this point was recorded as the corneal sensitivity threshold. Measurements were taken three times and averaged. Each experimental group included at least six mice.

### Corneal epithelial debridement healing

Mice were systemically anesthetized by intraperitoneal injection of 1.25% tribromoethanol (0.2 mL/10 g). Topical anesthesia was applied to the ocular surface using 0.5% proparacaine hydrochloride. A 2.5-mm corneal trephine was used to create a light central corneal impression. Under microscopic guidance, the corneal epithelium within the demarcated area was removed using an AlgerBrush II ring drill (Alger Inc., USA). At 0, 12, 24, and 36 h post-debridement, corneal epithelial defects were stained with 0.25% sodium fluorescein and examined under cobalt blue light with a slit lamp. Images were captured for documentation. Following imaging, the eyes were irrigated with saline and prophylactic ofloxacin ointment was applied to prevent infection. Corneal epithelial defect areas were quantified using Image J. Each experimental group included at least six mice.

### Whole-mount staining of corneal nerve fibers

One week after corneal epithelial debridement in diabetic mice, the eyeballs were collected and fixed in Zamboni fixative. Under microscopic guidance, intact corneal tissues were excised and divided into six wedges. The corneal tissues were subsequently permeabilized in blocking buffer containing 0.3% Triton X-100 and 10% goat serum. Samples were incubated overnight at 4 °C with antibody anti-β-tubulin III (657404, Biolegend, USA). Corneal nerve fiber density was examined under a laser scanning confocal microscope (Leica, Germany). Each experimental group included at least six mice.

### Subconjunctival injection

Prior to subconjunctival injection, mice were anesthetized systemically and topically. A 5-μL solution was administered to the inferior bulbar conjunctiva using a microinjector. For dual solution injections, separate injections were administered to superior and inferior bulbar conjunctiva. The injections were given at − 24, 0, and + 24 h relative to the time of injury. In successfully established type 1 diabetic mice, subconjunctival injections included: 2-DG (10 mM, Selleck Chemicals LLC, Houston, USA) or saline control. For BRG1 interventions, plasmids and empty vector controls were provided by Hanbio Biotechnology (Shanghai, China). Diabetic mice were randomly assigned to receive: (1) Saline, (2) BRG1 overexpression plasmid (pCMV-Smarca4-3flag-mcmv-EGFP; 200 ng/μL), (3) BRG1 knockdown plasmid (m-Smarca4 shRNA1; 200 ng/μL), (4) Empty vector plasmid (200 ng/μL). For PI3K/AKT pathway inhibition, mice received: LY294002 (10 μM, Beyotime Biotech Inc., Beijing, China) or negative control. Each experimental group contained at least three mice. All experiments were independently replicated to ensure data reliability.

### Western blot

Intact mouse trigeminal ganglion (TG) nerve tissues were lysed on ice using a mixed solution of radioimmunoprecipitation assay (RIPA) buffer and 1% phenylmethylsulfonyl fluoride (PMSF). An appropriate amount of 4 × protein loading buffer was added, and samples were boiled after equalization based on bicinchoninic acid (BCA) assay results. The resulting protein samples were separated by sodium dodecyl sulfate-polyacrylamide gel electrophoresis (SDS-PAGE) and transferred to polyvinylidene fluoride (PVDF) membranes. Membranes were blocked with 5% skim milk for 1 h, followed by overnight incubation with primary antibodies at 4 °C. The next day, membranes were thoroughly washed with Tris-buffered saline with 0.1% Tween 20 (TBST) solution and incubated with secondary antibodies at room temperature for 1 h. Protein bands were finally visualized using a chemiluminescence imaging system (Bio-Rad, USA). Each experiment was repeated at least three times. Each experimental group contained at least three mice. Primary antibodies used are listed in Supplementary Table S1.

### Immunofluorescence assay

Intact mouse TG tissues were embedded in optimal cutting temperature (OCT) (Tissue-Tek, Torrance, CA, USA) compound and sectioned into 5 μm thick frozen sections. Sections were fixed with 4% paraformaldehyde, permeabilized for 2 h with a mixed solution of 0.3% Triton X-100 and 10% goat serum, and incubated overnight at 4 °C with corresponding primary antibodies. Sections were then incubated with Alexa Fluor-conjugated secondary antibodies, followed by nuclear counterstaining with 4',6-diamidino-2-phenylindole (DAPI). Finally, fluorescence intensity was observed under an inverted fluorescence microscope (Leica, Germany). At least three replicates were performed per group to ensure data reliability. Each experimental group consisted of at least three mice. All primary antibodies used are listed in Supplementary Table S1.

### Quantitative real‑time polymerase chain reaction (qRT‑PCR)

Intact mouse TG tissues were dissected free of extraneous material under microscopic guidance and minced into fragments. Total ribonucleic acid (RNA) was extracted using TRIzol^™^ Reagent (Invitrogen, Waltham, MA, USA), reverse-transcribed into complementary DNA (cDNA) with 5X ALL-In-One RT MasterMix (Cat. No. G490, abm, Canada), and amplified using BlasTaq^™^ 2X qPCR MasterMix (Cat. No. G891/G892, abm, Canada). qRT-PCR was performed on an ABI 7500 Real-Time PCR System (Life Technologies, Singapore). β-actin served as the endogenous reference gene, and data were analyzed using the 2^−ΔΔCT^ method. Each experimental group comprised a minimum of three mice. Primers were custom-designed and synthesized by Sangon Biotech (Shanghai, China). Primer sequences used are listed in Supplementary Table S2.

### Lactate levels

Lactate concentration in mouse TG tissue was measured using the L-Lactate Dehydrogenase Assay Kit with WST-8 (L-LDH Assay Kit with WST-8) from Beyotime Biotech Inc. Tissue samples were prepared according to the manufacturer’s instructions. For every 10 mg of tissue, 100 μL of Lactate Assay Buffer was added. The tissue was then homogenized on ice (4 °C), followed by centrifugation. The resulting supernatant was collected and used for subsequent analysis. Prepared samples were transferred to a 96-well plate, mixed with the chromogenic working solution, and incubated at 37 °C for 30 min. The absorbance was measured at a wavelength of 450 nm. The lactate concentration in the samples was calculated based on the molecular weight of lactate. Each experimental group comprised a minimum of three mice.

### Statistical analyses

Experimental data were analyzed using GraphPad Prism 9.0.0 software (San Diego, CA, USA). Data are presented as mean ± standard error of the mean (SEM). Comparisons between groups were made using unpaired Student’s t-test or one-way analysis of variance (ANOVA), followed by Tukey's multiple comparisons test. All experiments were repeated at least three times. Statistical significance was defined as *P* < 0.05.

## Results

### Hyperglycemia induces corneal epithelial healing impairment, neurodegeneration, and neural glycolytic reprogramming

**Fig. 1:**
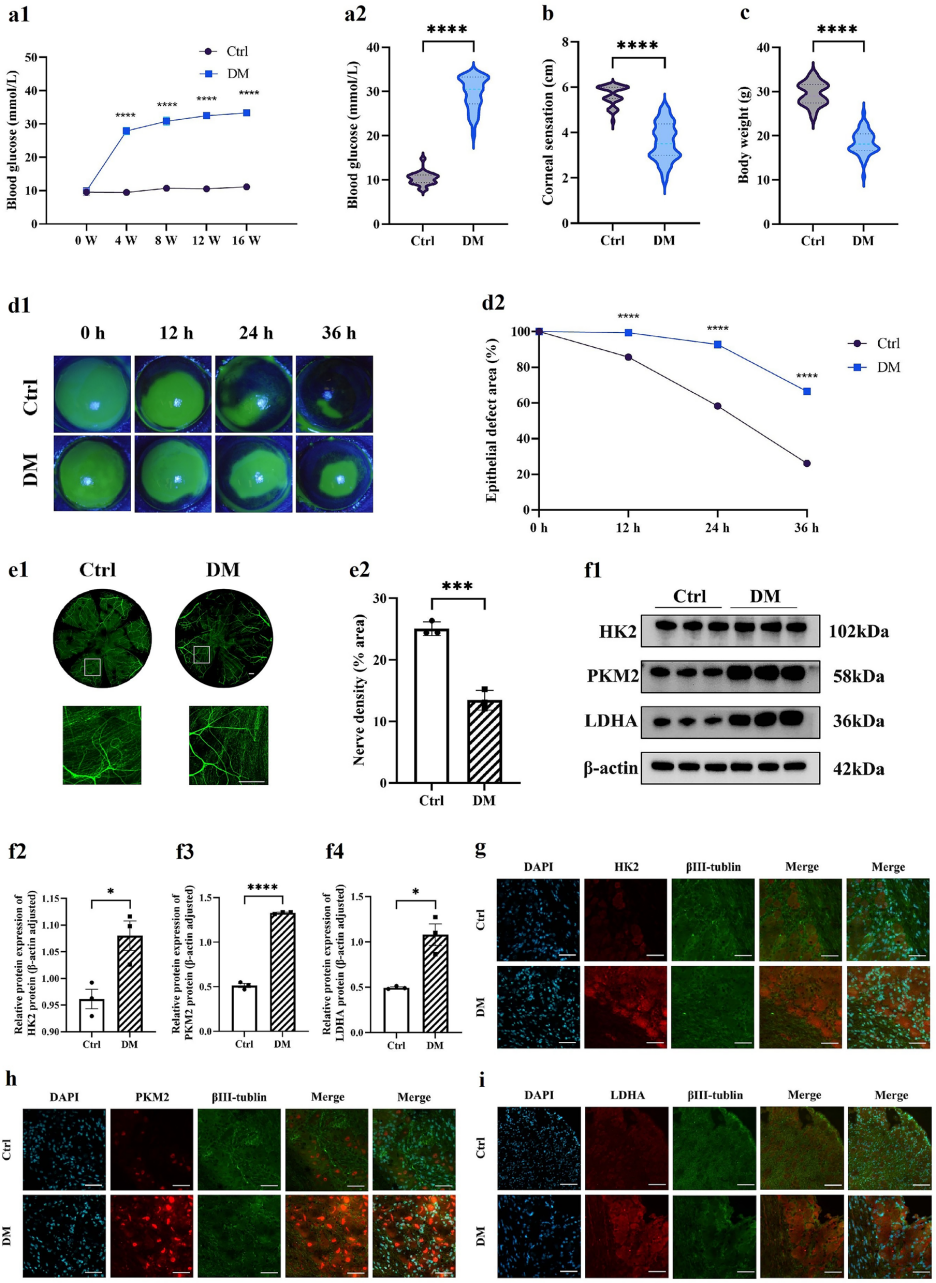
Hyperglycemia-induced delayed corneal epithelial healing and neurodegeneration. **a** Blood glucose levels in the diabetic (DM) group and the control (Ctrl) group after intraperitoneal injection. **a1** Blood glucose levels at weeks 0, 4, 8, 12, and 16. **a2** Blood glucose difference between DM and Ctrl mice at week 16. **b** Body weight difference at week 16 (DM vs. Ctrl). **c** Corneal sensitivity threshold (measured by Cochet-Bonnet aesthesiometer) at week 16. **d** Dynamic epithelial wound healing post-debridement (n = 6). **d1** Representative sodium fluorescein staining images at 0, 12, 24, and 36 h. **d2** Quantification of epithelial defect area (%). **e** Comparison of corneal whole-mount staining between DM and Ctrl mice (n = 6). **e1** Representative βIII-tubulin-stained images. **e2** Quantitative analysis of corneal nerve fiber density.** f** Protein expression of glycolytic enzymes in trigeminal nerve from Ctrl and DM mice (n = 3 per group). **f1** Representative Western blot bands for hexokinase 2 (HK2), pyruvate kinase M2 (PKM2), lactate dehydrogenase A (LDHA), and β-actin. Quantification of HK2 (**f2**), PKM2 (**f3**), and LDHA (**f4**) expression levels normalized to β-actin. Immunofluorescence intensity comparison of HK2 (**g**), PKM2 (**h**), and LDHA (**i**) in trigeminal nerve (n = 3 per group). Statistical differences were determined by Student's t-test. Scale bars: 50 μm. *P < 0.05; ***P < 0.001; ****P < 0.0001. DAPI, 4',6-diamidino-2-phenylindole

Compared to age-matched control mice, diabetic model mice exhibited significantly elevated blood glucose (*P* < 0.05) and reduced body weight (*P* < 0.05) (Fig. 1a1, a2, b), consistent with type 1 diabetes characteristics and confirming successful modeling. In epithelial wound healing assays, sodium fluorescein staining revealed significantly delayed wound closure in diabetic mice at 12, 24, and 36 h post-injury versus controls (Fig. 1d1, d2), indicating impaired epithelial regenerative capacity under hyperglycemia. Concurrently, corneal sensitivity was significantly reduced in the diabetic group (Fig. 1c). Confocal microscopy confirmed decreased corneal nerve density in diabetic mice, with high-magnification images demonstrating preferential loss of the subbasal nerve plexus (Fig. 1e1, e2), indicative of regenerative impairment of corneal nerves in diabetes.

To investigate the association between DCN and glycolysis, we compared levels of glycolytic rate-limiting enzymes in trigeminal nerve of type 1 diabetic mice versus non-diabetic controls. Results demonstrated significantly elevated protein levels of HK2, pyruvate kinase M2 (PKM2), and LDHA in trigeminal nerve of diabetic mice (Fig. 1f). To verify neuronal localization, immunofluorescence co-staining was performed using antibodies against glycolytic enzymes and βIII-tubulin (pan-neuronal marker). All three enzymes exhibited co-localization with neuronal structures. HK2 and LDHA exhibited predominant cytoplasmic localization (primary site of glycolytic reactions), suggesting upregulation of glycolytic enzymes. Notably, PKM2 demonstrated dual cytoplasmic and nuclear distribution (Fig. 1g–i).

In summary, hyperglycemia drives delayed corneal epithelial healing and neurodegenerative alterations through induced neural metabolic reprogramming.

### Enhanced glycolysis exacerbates impairment of corneal wound healing in diabetes

**Fig. 2:**
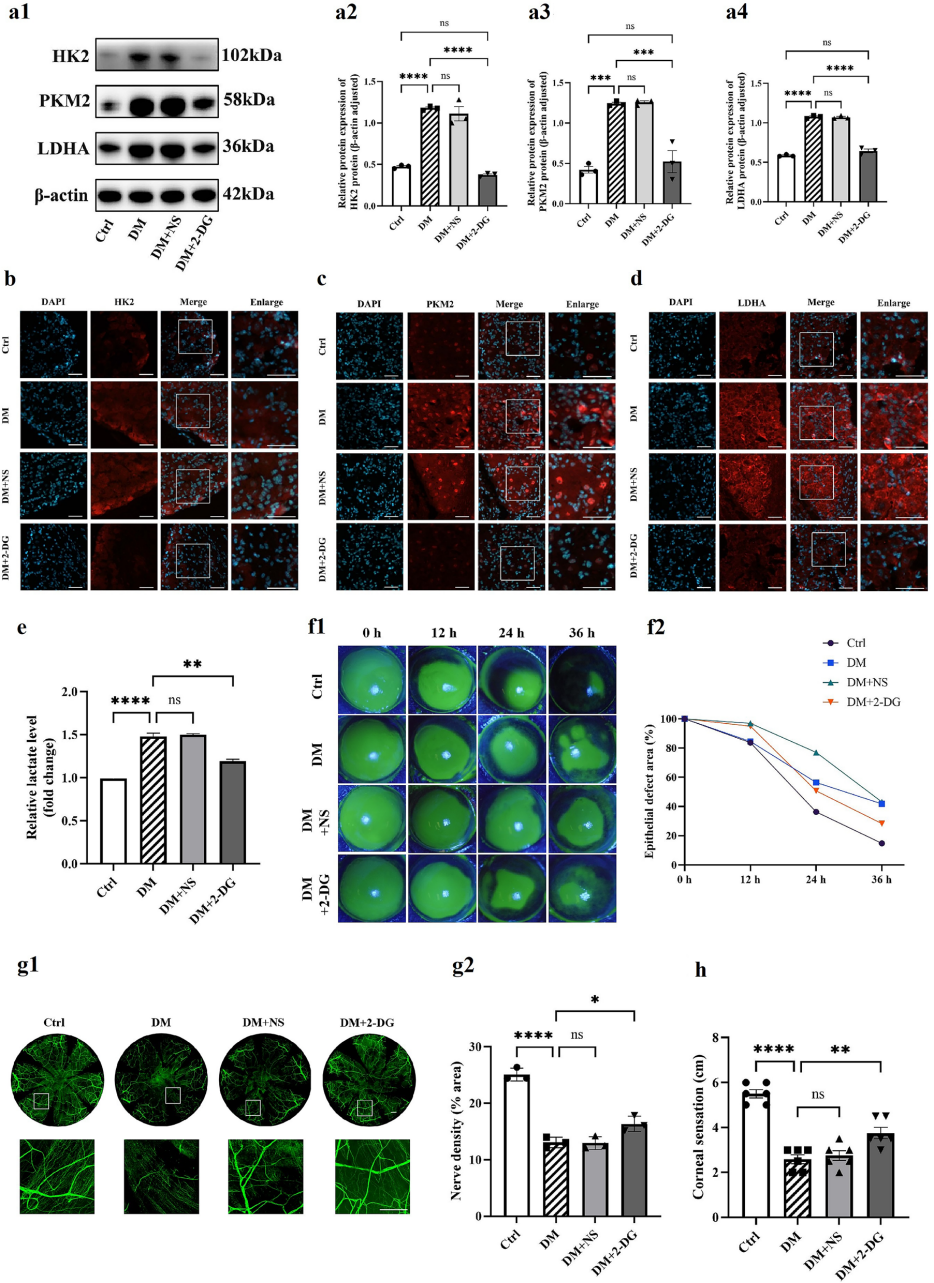
Glycolysis inhibition promotes diabetic corneal epithelial healing and nerve regeneration. **a** Western blot analysis of glycolytic enzymes in trigeminal nerve. Groups: control (Ctrl), diabetic (DM), subconjunctival saline injection (DM + NS), subconjunctival 2-DG injection (DM + 2-DG) (n = 3 per group). **a1** Representative Western blot bands for hexokinase 2 (HK2), pyruvate kinase M2 (PKM2), and lactate dehydrogenase A (LDHA). Quantification of HK2 (**a2)**, PKM2 (**a3**), and LDHA (**a4**) expression normalized to β-actin. Immunofluorescence intensity of HK2 (**b**), PKM2 (**c**), and LDHA (**d**) in trigeminal nerve (n = 3 per group). **e** Lactate levels (fold change vs. Ctrl) in trigeminal nerve (n = 6). **f** Dynamic corneal epithelial healing post-debridement (n = 6). **f1** Representative sodium fluorescein staining images at 0, 12, 24, and 36 h. **f2** Quantified epithelial defect area (%). **g** Corneal nerve regeneration analysis (n = 6). **g1** Representative βIII-tubulin-stained whole-mount images. **g2** Corneal nerve fiber density (% area). **h** Corneal sensitivity threshold across groups (n = 6). Statistical analysis was performed by one-way ANOVA followed by Tukey's multiple comparisons test. Scale bars: 50 μm. ns, not significant; *P < 0.05; **P < 0.01; ***P < 0.001; ****P < 0.0001. DAPI, 4',6-diamidino-2-phenylindole

To validate the role of elevated glycolytic flux in DCN, we administered 2-deoxy-D-glucose (2-DG), a non-metabolizable glucose analog, via subconjunctival injection. Following one week of intervention, Western blot and immunofluorescence analyses revealed a significant reduction in the expression of key rate-limiting glycolytic enzymes (HK2, PKM2, LDHA) within the trigeminal nerve of 2-DG-treated mice. This decrease was accompanied by a decline in lactate levels in the ganglia, demonstrating that 2-DG effectively inhibited glycolysis in trigeminal neurons and alleviated glycolysis-derived microenvironment acidification (Fig. 2a–e).

Sodium fluorescein staining demonstrated accelerated corneal epithelial wound healing in 2-DG-treated diabetic mice compared to untreated diabetic controls, though restoration remained incomplete relative to non-diabetic mice (Fig. 2f). Confocal microscopy and corneal sensitivity assessment revealed that compared to the diabetic control group, the 2-DG group exhibited increased subbasal nerve plexus density and elevated corneal sensitivity (Fig. 2g, h). This indicates that glycolytic inhibition positively contributes to diabetic corneal epithelial repair and nerve regeneration, thereby partially delaying DCN progression. However, it failed to restore the mice to the normal levels observed in non-diabetic mice.

Collectively, augmented glycolytic flux exacerbates impairments in corneal wound healing in diabetic mice, while its inhibition improves epithelial repair and partially reverses the impairments in nerve regeneration, albeit without achieving full restoration.

### Upregulation of BRG1 and its glycolytic regulatory role in trigeminal tissues

**Fig. 3:**
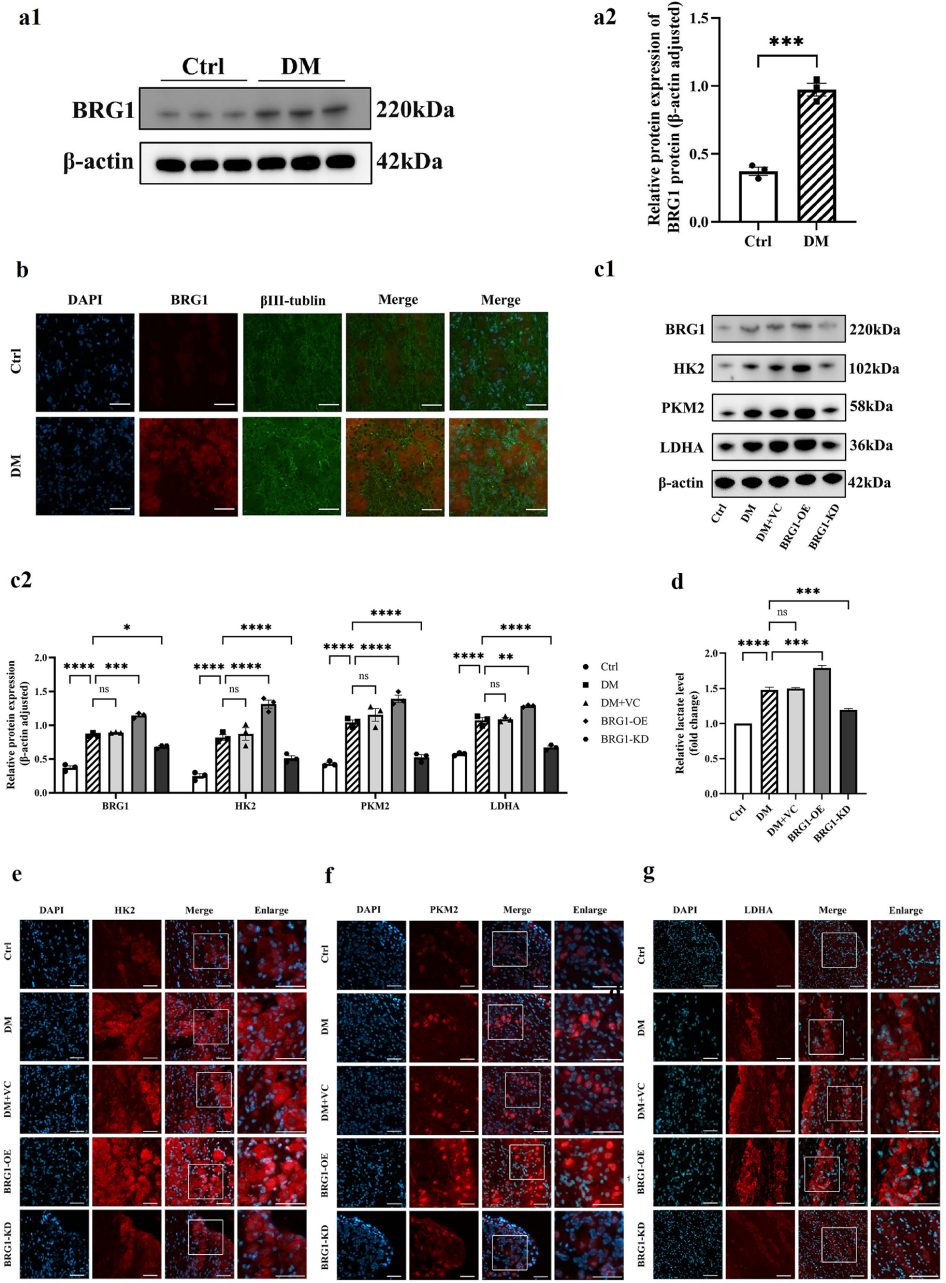
BRG1 is a master regulator of glycolytic flux and lactate production in diabetic trigeminal tissues. **a** Protein expression of Brahma-related gene 1 (BRG1) in control (Ctrl) and diabetic (DM) mice. **a1** Representative Western blot bands. **a2** Quantification of BRG1 levels normalized to β-actin. **b** Co-localization of BRG1 with βIII-tubulin⁺ neurons in trigeminal nerve (immunofluorescence). **c** Western blot analysis of BRG1 and glycolytic enzymes in trigeminal nerve (n = 3 per group). Groups: Ctrl, DM, subconjunctival empty vector control (DM + VC), subconjunctival pCMV-Smarca4-3flag-mcmv-EGFP (BRG1-OE), subconjunctival m-Smarca4 shRNA1 (BRG1-KD). **c1** Western blot bands for BRG1, hexokinase 2 (HK2), pyruvate kinase M2 (PKM2), and lactate dehydrogenase A (LDHA). **c2** Protein expression levels of BRG1, HK2, PKM2, and LDHA, normalized to β-actin. **d** Lactate levels in trigeminal nerve across groups. Immunofluorescence intensity of HK2 (**e**), PKM2 (**f**), and LDHA (**g**) in trigeminal nerve (n = 3 per group). Statistical analysis was performed by one-way ANOVA followed by Tukey's multiple comparisons test. Scale bars: 50 μm. ns, not significant; *P < 0.05; **P < 0.01; ***P < 0.001; ****P < 0.0001. DAPI, 4',6-diamidino-2-phenylindole

Quantitative Western blot analysis revealed a significant elevation of BRG1 protein levels in the trigeminal nerve of diabetic mice compared to normoglycemic controls, indicating hyperglycemia-induced upregulation of neuronal BRG1 expression (Fig. 3a). Immunofluorescence staining further demonstrated predominant cytoplasmic localization of BRG1, which colocalized with key glycolytic enzymes (Fig. 3b). To define the impact of BRG1 on glycolysis within the diabetic trigeminal nerve, we first modulated BRG1 expression via subconjunctival injection (administering either pCMV-Smarca4-3FLAG-mCMV-EGFP for overexpression or m-Smarca4 shRNA1 for knockdown). Western blot analysis revealed that, compared to the empty vector control group, BRG1 overexpression significantly elevated BRG1 protein levels in the diabetic trigeminal nerve, whereas BRG1 knockdown markedly reduced BRG1 expression (Fig. 3c).

Concomitantly, BRG1 overexpression significantly upregulated protein levels of critical glycolytic enzymes (HK2, PKM2, LDHA) compared to diabetic controls. Immunofluorescence analysis showed enhanced LDHA fluorescence intensity in neuronal cells, alongside elevated lactate levels in trigeminal nerve. In stark contrast, BRG1 knockdown broadly suppressed glycolytic enzyme expression, markedly attenuated neuronal LDHA fluorescence, and reduced lactate accumulation in trigeminal nerve (Fig. 3d–g).

These findings indicate that hyperglycemia mediates the upregulation of BRG1 in diabetic trigeminal nerve, while BRG1 orchestrates glycolytic reprogramming by enhancing the expression of glycolytic enzymes.

### Pathway screening implicates PI3K/AKT in BRG1-dependent glycolytic regulation of DCN

To delineate the specific pathway through which BRG1 regulates glycolysis, we conducted a comprehensive signaling pathway screen. BRG1 modulates multiple metabolic pathways through epigenetic regulation, thereby influencing the pathogenesis of various diseases (Supplementary Table S3). Through systematic literature mining, we identified four candidate pathways intersecting BRG1 and glycolytic regulation: PI3K/AKT, Wingless-type/integrated-β-catenin (Wnt/β-catenin) [24], cGAS/STING [25], and nuclear factor erythroid 2-related factor 2/heme oxygenase-1 (Nrf2/HO-1) [18].

**Fig. 4:**
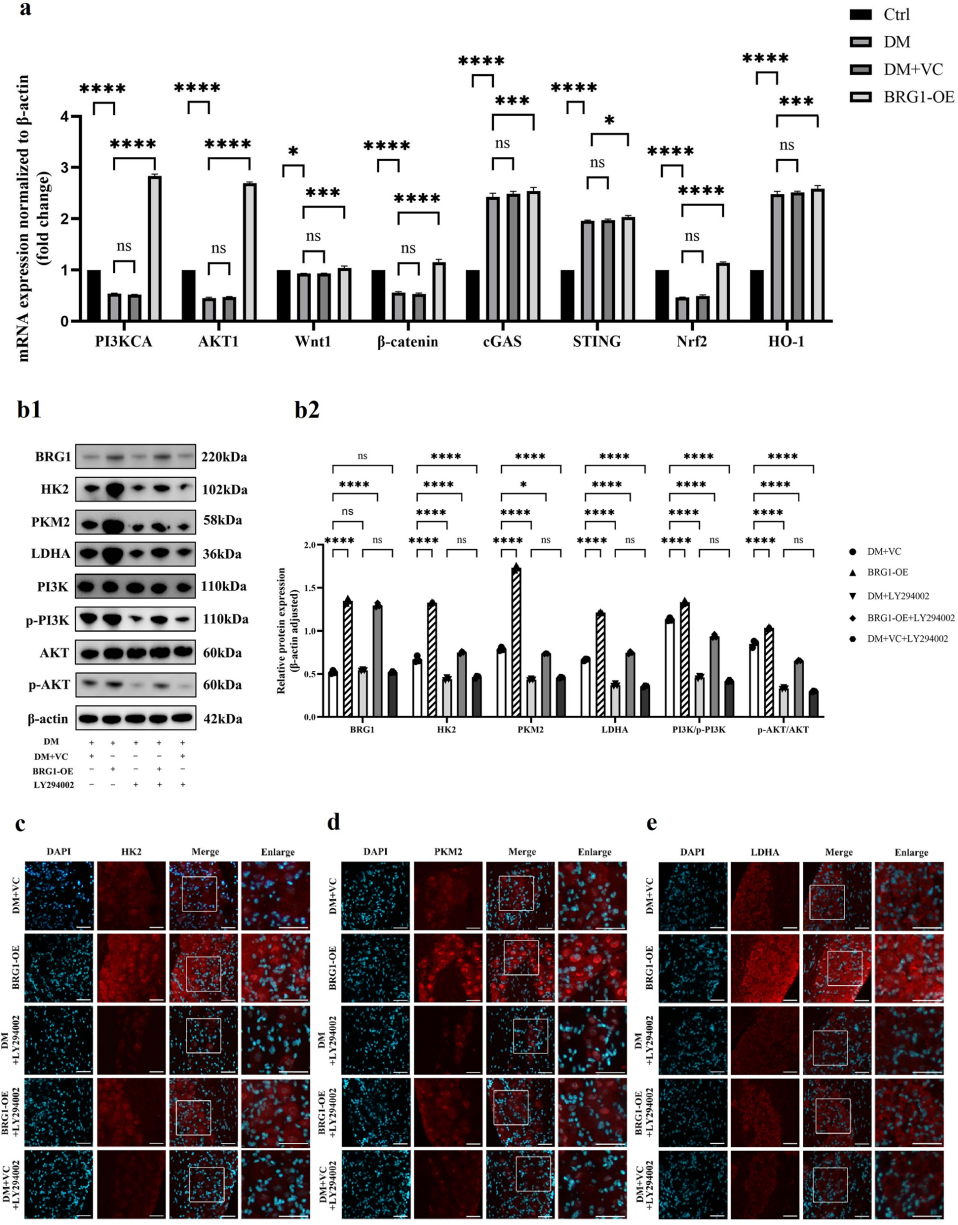
BRG1 drives glycolytic activation via the PI3K/AKT Pathway. **a** mRNA expression of signaling pathways by quantitative real-time polymerase chain reaction (qRT-PCR). Targets: phosphatidylinositol 3-kinase/protein kinase B (PI3K/AKT), Wnt/β-catenin, cGAS/STING, Nrf2/HO-1. **b** Western blot analysis of glycolytic enzymes and PI3K/AKT components post-BRG1 overexpression with subconjunctival LY294002 injection. **b1** Representative Western blot bands for Brahma-related gene 1 (BRG1), hexokinase 2 (HK2), pyruvate kinase M2 (PKM2), lactate dehydrogenase A (LDHA), PI3K, phospho-PI3K, AKT, and phospho-AKT (Ser473). **b2** Quantification of protein expression normalized to β-actin. Immunofluorescence intensity of HK2 (**c**), PKM2 (**d**), and LDHA (**e**) in trigeminal nerve (n = 3 per group). Statistical analysis was performed by one-way ANOVA followed by Tukey's multiple comparisons test. Scale bars: 50 μm. ns, not significant; *P < 0.05; **P < 0.01; ***P < 0.001; ****P < 0.0001. Ctrl, control; DM, diabetic; DM + VC, subconjunctival empty vector control; DM + LY294002, subconjunctival LY294002; BRG1-0E + LY294002, subconjunctival injection of pCMV-Smarca4-3flag-mcmv-EGFP + LY294002; DM + VC + LY294002, subconjunctival empty vector control + LY294002; BRG1-OE, subconjunctival pCMV-Smarca4-3flag-mcmv-EGFP; DAPI, 4',6-diamidino-2-phenylindole

qRT-PCR analysis revealed distinct pathway alterations in diabetic trigeminal nerve compared to normoglycemic controls: the PI3K/AKT, Wnt/β-catenin, and Nrf2/HO-1 pathways exhibited significant messenger RNA (mRNA) downregulation. Conversely, components of the cGAS/STING pathway were upregulated. Crucially, BRG1 overexpression universally elevated mRNA levels across all four pathways. The most pronounced upregulation occurred in PI3K/AKT signaling transduction (Fig. 4a). The PI3K/AKT signaling pathway serves as a central hub for cellular stress responses and is extensively implicated in diabetic complications. This preferential potentiation of PI3K/AKT, coupled with its established role in transactivating glycolytic enzymes (e.g., GLUT1/HK2/LDHA [26]), suggests that PI3K/AKT acts as the primary conduit for BRG1-mediated glycolytic control in diabetic neuropathy. Hence, we propose that PI3K/AKT is the specific route through which BRG1 orchestrates glycolytic reprogramming.

### BRG1-driven glycolytic activation requires PI3K/AKT signaling in diabetic trigeminal tissues

To validate PI3K/AKT as the specific pathway through which BRG1 orchestrates glycolytic reprogramming, we inhibited this axis via subconjunctival injection of LY294002 (a PI3K allosteric inhibitor).

As shown in Fig. 4b, LY294002 treatment did not alter BRG1 protein expression. Following significant suppression of PI3K phosphorylation by LY294002, protein levels of key glycolytic enzymes (HK2, PKM2, LDHA) were markedly downregulated, demonstrating PI3K/AKT-mediated control of glycolytic flux. Critically, when LY294002 was co-administered with BRG1 overexpression, the pro-glycolytic effects of BRG1 were reversed: HK2, PKM2, and LDHA expression were significantly reduced compared to BRG1 overexpression alone (Fig. 4b). This attenuation was further validated by diminished immunofluorescence intensity of these enzymes (Fig. 4c–e). Mechanistically, LY294002 interrupts BRG1-initiated signaling by blocking PI3K kinase activity, thereby suppressing downstream glycolytic effector expression. These results conclusively establish that BRG1’s pro-glycolytic function requires PI3K/AKT signaling transduction.

### Activation of the BRG1-PI3K/AKT axis exacerbates impairment of corneal wound healing in diabetes by disrupting metabolic homeostasis

To delineate BRG1’s role in DCN progression, we systematically modulated BRG1 expression (overexpression/knockdown) combined with PI3K inhibitor LY294002 intervention, assessing corneal epithelial repair kinetics and neurostructural/functional parameters (nerve fiber density and corneal sensitivity).

**Fig. 5:**
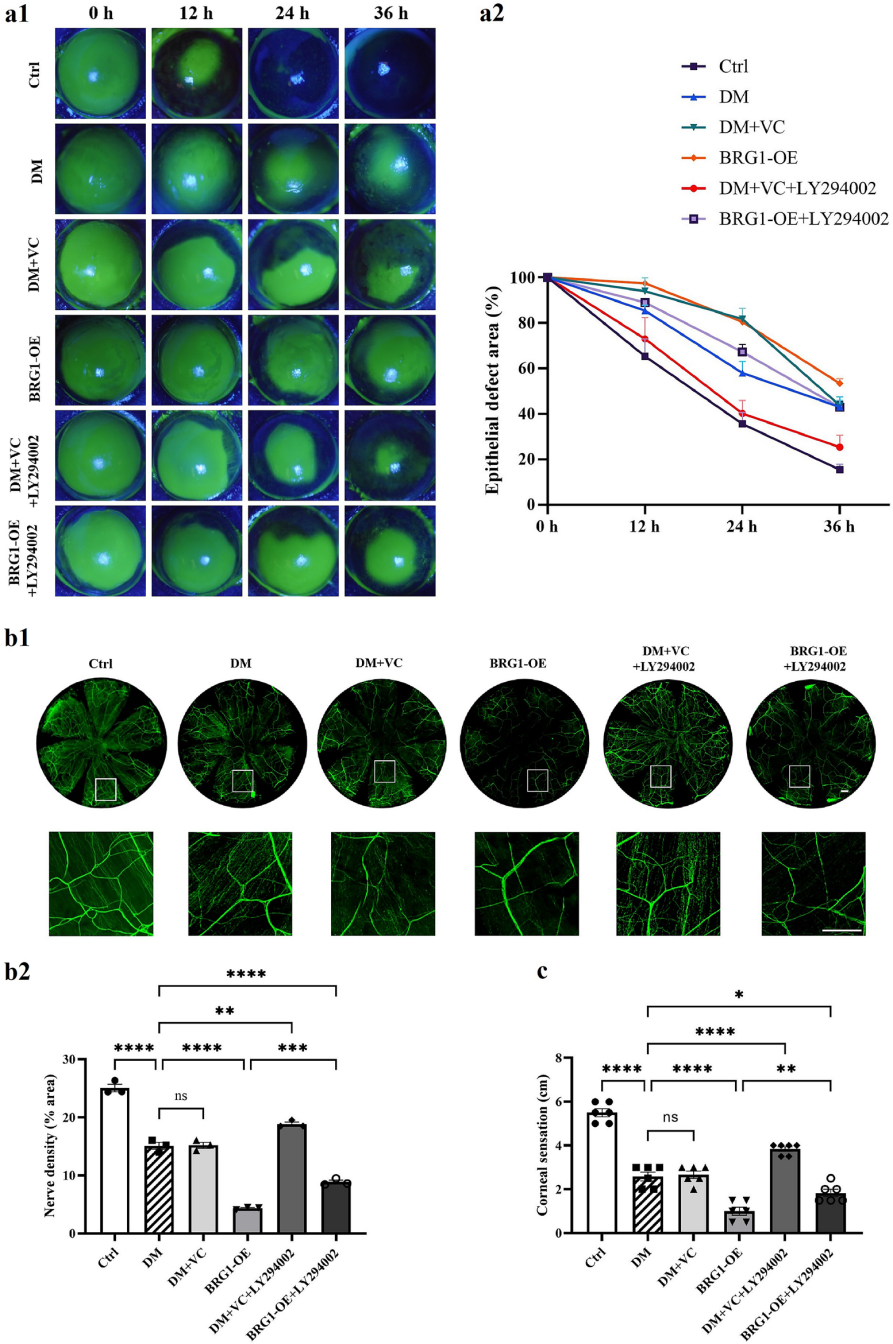
BRG1 impairs corneal epithelial healing and nerve regeneration via the PI3K/AKT pathway. **a** Dynamic corneal epithelial healing post-debridement. Groups: control (Ctrl), diabetic (DM), subconjunctival empty vector control (DM + VC), subconjunctival pCMV-Smarca4-3flag-mcmv-EGFP (BRG1-OE), subconjunctival injection of empty vector plasmid + LY294002 (DM + VC + LY294002), subconjunctival injection of pCMV-Smarca4-3flag-mcmv-EGFP + LY294002 (BRG1-OE + LY294002). **a1** Representative sodium fluorescein staining images at 0, 12, 24, and 36 h. **a2** Quantified epithelial defect area (%). **b** Corneal nerve regeneration analysis (n = 6). **b1** Representative βIII-tubulin-stained whole-mount images. **b2** Quantitative analysis of corneal nerve fiber density.** c** Corneal sensitivity threshold across groups (n = 6). Statistical analysis was performed by one-way ANOVA followed by Tukey's multiple comparisons test. Scale bars: 50 μm. ns, not significant; *P < 0.05; **P < 0.01; ***P < 0.001; ****P < 0.0001

Results demonstrated that compared to untreated diabetic mice, diabetic mice receiving subconjunctival BRG1 overexpression exhibited significantly reduced corneal nerve density, diminished corneal sensitivity, and delayed corneal epithelial healing rates. The PI3K inhibitor LY294002 reversed these BRG1-induced impairments in post-injury epithelial repair, nerve regeneration, and corneal sensitivity in the diabetic cornea (Fig. 5a–c).

## Discussion

**Fig. 6:**
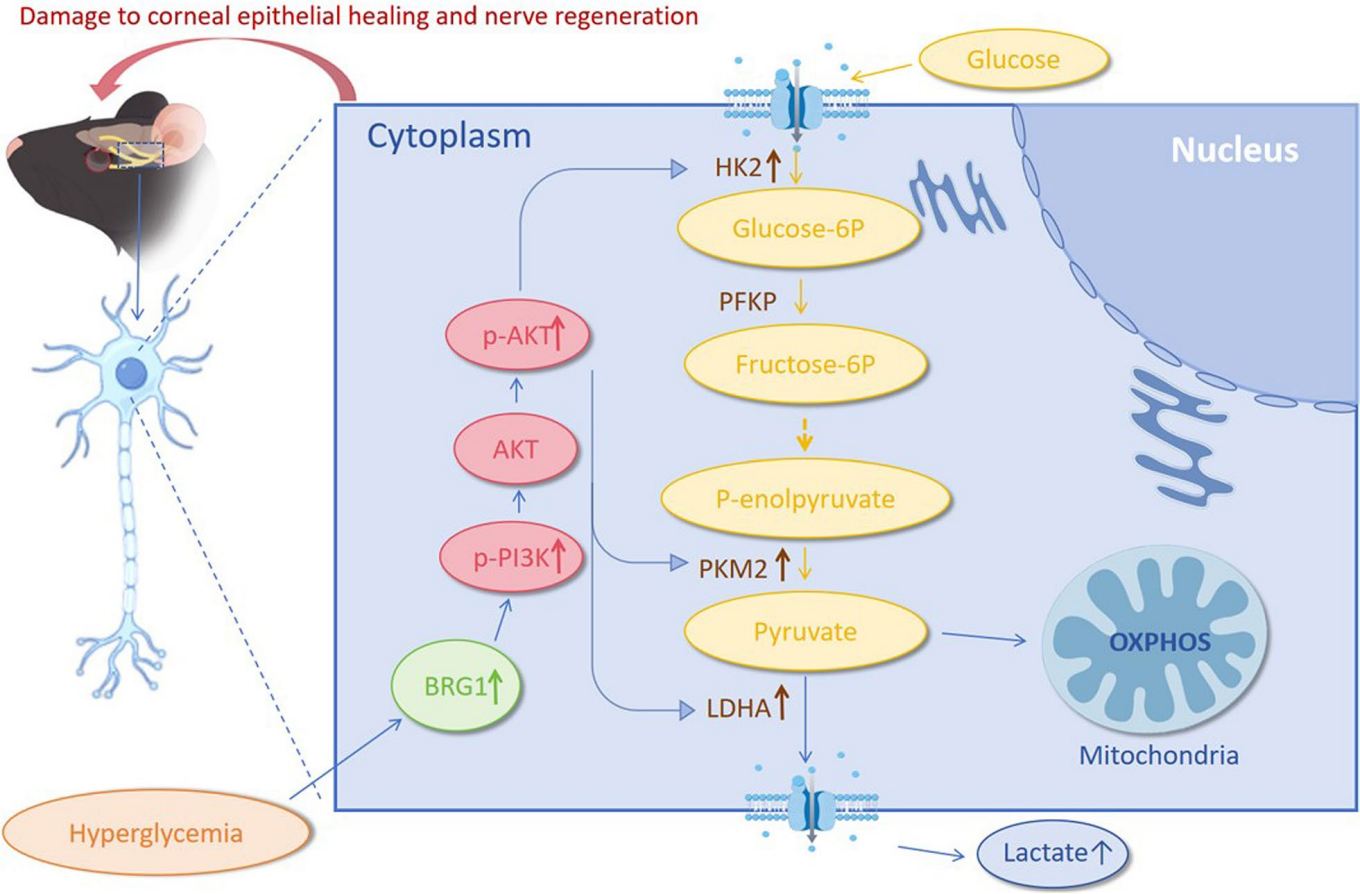
Schematic of the underlying mechanism: BRG1 impairs corneal wound healing and neural regeneration in diabetic mice via the PI3K/AKT-Glycolysis axis. HK2, hexokinase 2; PKM2, pyruvate kinase M2; LDHA, lactate dehydrogenase A; BRG1, Brahma-related gene 1; PI3K, phosphatidylinositol 3-kinase; AKT, protein kinase B

Pathological alterations in corneal nerves, as a complication of diabetes, have garnered increasing attention. The development of early prevention and therapeutic strategies for DCN represents an urgent clinical challenge. In this study, we unveiled the regulatory role of glycolytic flux in DCN pathogenesis and report for the first time that BRG1 modulates DCN through PI3K/AKT-mediated glycolytic control. The interaction between them is depicted in Fig. 6.

Prior studies demonstrated that chronic hyperglycemia disrupts neural fiber integrity and impairs nerve regeneration via multiple pathways, ultimately leading to DCN [3]. Glycolysis serves as a critical energy source for neurons and is a focal point in diabetic neuropathy research [27]. Evidence indicates that hyperglycemia induces a metabolic reprogramming toward glycolytic dependency in neural tissues, while pathological accumulation of lactate, the end-product of glycolysis, directly contributes to neuronal damage and neuropathological progression [28]. Consistent with these observations, our data reveal upregulated expression of glycolytic genes in diabetic mice. Neuronal immunofluorescence analysis demonstrated exclusive cytoplasmic localization of HK2 and LDHA, whereas PKM2 exhibited dual cytoplasmic and nuclear distribution, suggesting its potential dual roles in metabolic regulation and nuclear transcriptional functions. Notably, therapeutic inhibition of glycolytic flux with 2-DG attenuated and partially reversed DCN progression. This key finding not only confirms that glycolytic reprogramming is a core pathological mechanism in this disease but also highlights the therapeutic potential of targeting glycolysis. Although 2-DG did not fully restore corneal integrity to non-diabetic levels, its significant partial efficacy provides a strong experimental rationale for developing more specific or potent glycolytic inhibitors as a treatment strategy for DCN.

This study reveals for the first time that hyperglycemia-induced BRG1 upregulation drives glycolytic reprogramming via epigenetic remodeling in DCN, thereby exacerbating neurodegeneration and repair impairment. This discovery expands the functional understanding of BRG1 in metabolic disorders: it acts not merely as an environmental stress “sensor” but as an active “executor” orchestrating metabolic dysregulation. Our data demonstrates synchronized upregulation of BRG1 and glycolytic rate-limiting enzymes (HK2, PKM2, LDHA) in diabetic trigeminal tissues. BRG1 overexpression significantly suppressed corneal epithelial repair and accelerated neurodegeneration, which is consistent with prior reports. Functional interventions confirmed BRG1’s pathogenic role, as its overexpression delayed epithelial healing, reduced nerve density, and diminished corneal sensitivity, paralleled by elevated glycolytic enzyme expression. While BRG1 knockdown partially reversed damage, it failed to restore parameters to non-diabetic levels. This indicates that although BRG1 inhibition provides protection, its efficacy may be constrained by parallel hyperglycemia-driven pathways such as advanced glycation end-product (AGE) accumulation or oxidative stress, thereby preventing complete metabolic compensation. These findings suggest that BRG1 acts as a collaborative pathogenic factor within a complex metabolic dysregulation network, a hypothesis requiring further experimental validation.

The PI3K/AKT signaling pathway serves as a central regulatory hub for glycolytic reprogramming. Its role in activating key glycolytic enzymes (HK2, PKM2) and lactate dehydrogenase (LDHA) in cancer has been extensively documented [26, 29–32]. PI3K/AKT signaling also contributes to neural cell proliferation [33] and is expressed in both neurons and Schwann cells, where it modulates nerve regeneration and repair processes [34–36]. Our work identifies this pathway as the dominant conduit for glycolytic reprogramming in DCN: PI3K inhibition by LY294002 significantly suppressed HK2, PKM2, and LDHA expression. Furthermore, BRG1 has been implicated in PI3K/AKT regulation across disease contexts [22, 37–39]. Here, BRG1 overexpression markedly upregulated PI3K/AKT pathway transcripts, while LY294002-mediated PI3K inhibition did not alter BRG1 expression, confirming BRG1’s upstream position in unidirectional regulation. However, the PI3K/AKT pathway can be activated by many upstream signals, such as common growth factor receptors, Toll-like receptors, and glucose transporters like GLUT1. This complexity could raise questions about how specifically BRG1 regulates PI3K/AKT. However, key evidence from our study supports the specific role of BRG1. First, all our experiments were done in a stable diabetic environment. In this setting, changing BRG1 levels was sufficient to significantly alter PI3K/AKT signaling and downstream glycolysis. Second, and most importantly, when we used the inhibitor LY294002 to block PI3K/AKT, it reversed the harmful effects caused by BRG1 overexpression, without changing BRG1 itself. This clearly shows a one-way relationship: BRG1 → PI3K/AKT → Glycolysis. Therefore, we conclude that BRG1 is a key and specific epigenetic driver that coordinates PI3K/AKT-mediated metabolic reprogramming.

This study has limitations. This study is primarily focused on a type 1 diabetic model. Given the considerable incidence of human DCN in type 2 diabetes, our future work will employ type 2 diabetic models (e.g., db/db mice) and human data to further validate the role of the BRG1-PI3K/AKT axis in human DCN. Furthermore, given that BRG1-targeted monotherapy incompletely reverses damage, future studies should explore combinatorial targeting strategies to evaluate potential synergistic therapeutic effects. Additionally, while LY294002, a broad-spectrum PI3K/AKT pathway inhibitor, is recognized for its relatively high selectivity, it is also documented to inhibit other kinases such as casein kinase 2 (CK2) [40, 41]. Building upon the foundation of this study, future research should aim to achieve therapeutic goals while minimizing adverse effects by implementing improved strategies. These may include employing pathway-specific targeting approaches, developing or identifying more potent and selective inhibitors, and designing tissue-specific or cell-specific delivery systems. Such advancements would enable localized and specific modulation of the pathway, thereby minimizing off-target effects and systemic impact.

Collectively, we demonstrate that BRG1 orchestrates glycolytic reprogramming through PI3K/AKT activation, establishing its role as a critical upstream master regulator of DCN pathogenesis. Future translational efforts should refine targeted delivery strategies to maximize therapeutic efficacy while minimizing metabolic perturbation.

## Conclusions

Our findings demonstrate that enhanced glycolysis is a central driver of impaired corneal re-epithelialization and neuroregeneration deficits in diabetic wound healing. Critically, BRG1 exacerbates these pathological processes by augmenting glycolytic flux through activation of the PI3K/AKT signaling pathway, thereby contributing to delayed epithelial repair and impaired nerve regeneration.

## Supplementary Information

Supplementary material 1.

## Data Availability

All data are included in the manuscript and supplementary materials. The resources during the current study are available upon reasonable request.

## Abbreviations

STZ: Streptozotocin
DCN: Diabetic corneal neuropathy
TG: Trigeminal ganglion
H3K27me3: Histone H3 lysine 27 trimethylation
H3K9me3: Histone H3 lysine 9 trimethylation
H3K27ac: Histone H3 lysine 27 acetylation
H3K14ac: Histone H3 lysine 14 acetylation
qRT-PCR: Quantitative real-time polymerase chain reaction
PI3K/AKT: Phosphatidylinositol 3-kinase/protein kinase B
BRG1: Brahma-related gene 1
2-DG: 2-Deoxy-D-glucose
CGRP: Calcitonin gene-related peptide
EGF: Epidermal growth factor
ATP: Adenosine triphosphate
AMPK: Adenosine monophosphate-activated protein kinase
cGAS/STING: Cyclic GMP-AMP synthase/stimulator of interferon genes
GLUT1: Glucose transporter 1
HK2: Hexokinase 2
PKM2: Pyruvate kinase M2
LDHA: Lactate dehydrogenase A
SWI/SNF: Switch independent/sucrose non-fermentable
PFK-1: Phosphofructokinase-1
PMSF: Phenylmethylsulfonyl fluoride
TBST: Tris-buffered saline with Tween 20
OCT: Optimal cutting temperature
DAPI: 4',6-diamidino-2-phenylindole
RNA: Ribonucleic acid
mRNA: Messenger RNA
Wnt/β-catenin: Wingless-type/integrated-β-catenin
Nrf2/HO-1: Nuclear factor erythroid 2-related factor 2/heme oxygenase-1
AGE: Advanced glycation end-product
CK2: Casein kinase 2
