# Supplementary material for: BRG1 orchestrates diabetic corneal neuropathy via PI3K/AKT-mediated glycolytic reprogramming
Source: Eye Vis (Lond). 2026 Feb 1;13:5. doi: 10.1186/s40662-026-00474-4 (PMC12861070; doi:10.1186/s40662-026-00474-4)
Supplement: Supplementary file 1 — Supplementary material 1. [file 40662_2026_474_MOESM1_ESM.docx]

**Supplemental Table S1. Primary antibodies for Western blot and immunofluorescence**

| Antibody | Catalog number | Manufacturers |
| --- | --- | --- |
| anti-HK2 | 2867 | CST, USA |
| anti-Brg1 | 49360 | CST, USA |
| anti-AKT | 9272 | CST, USA |
| anti-pSer472 AKT | 4060 | CST, USA |
| anti-PI3K | 4249 | CST, USA |
| anti-Phospho-PI3K | 13857 | CST, USA |
| anti-β-actin | ab8226 | Abcam, USA |
| anti-PKM2 | 15822 | Proteintech, Wuhan, China |
| anti-LDHA | 21799 | Proteintech, Wuhan, China |

CST denotes Cell Signaling Technology, Inc.

**Supplemental Table S2. Primer sequences for conventional quantitative real-time polymerase chain reaction (qRT-PCR)**

| Gene | Forward primer (5′-3′) | Reverse primer (5′-3′) |
| --- | --- | --- |
| PI3KCA | GGACTGTGTGGGTCTCATCG | TCTCGCCCTTGTTCTTGTCC |
| AKT1 | CCTCAAGAACGATGGCACCT | TGCAGGCAGCGGATGATAAA |
| cGAS | GGGCACAAAAGTGAGGACCA | CGCCAGGTCTCTCCTTGAAA |
| STING | ACTCTTCTGCCGGACACTTG | TTCCGTCTGTGGGTTCTTGG |
| Wnt1 | TGACAACATCGATTTTGGTCGC | CCTCGTTGTTGTGAAGGTTCATG |
| β-catenin | AGACAGCTCGTTGTACTGCT | GTGTCGTGATGGCGTAGAAC |
| Nrf2 | CACATCCAGTCAGAAACCAGTGG | GGAATGTCTGCGCCAAAAGCTG |
| HO-1 | TGCTCGCATGAACACTCTG | TCCTCTGTCAGCAGTGCCT |
| β-actin | GTACCACCATGTACCCAGGC | AACGCAGCTCAGTAACAGTCC |

**Supplemental Table S3. Pathway screening literature summary**

| Study | Annotation | Main conclusion |
| --- | --- | --- |
| Xuan et al. 2024 [1] | PI3K/AKT | BRG1 improves reprogramming efficiency by enhancing glycolytic  metabolism. |
| Wenjing et al. 2018 [2] | PI3K/AKT/mTOR | Brg1 aggravates airway inflammation in asthma via inhibition of the PI3K/Akt/mTOR pathway. |
| Dong et al. 2022 [3] | PI3K/AKT/Wnt/β-catenin | ROS/PI3K/Akt and Wnt/β-catenin signalings activate HIF-1α-induced metabolic reprogramming to impart 5-fluorouracil resistance in colorectal cancer. |
| Wangqiu et al. 2021 [4] | Wnt/β-catenin | Brahma-related gene-1 promotes tubular senescence and renal fibrosis through Wnt/β-catenin/autophagy axis. |
| Chen et al. 2025 [5] | cGAS/STING | BRG1 deficiency promotes cardiomyocyte inflammation and apoptosis by activating the cGAS-STING signaling in diabetic cardiomyopathy. |
| Mian et al. 2017 [6] | Nrf2/HO-1 | Brg1-mediated Nrf2/HO-1 pathway activation alleviates hepatic ischemia–reperfusion injury. |
| Sun et al. 2020 [7] | Nrf2/HO-1 | Upregulation of heme oxygenase-1 by Brahma-related gene 1 through Nrf2 signaling confers protective effect against high glucose-induced oxidative damage of retinal ganglion cells. |

**References**

1 Ren X, Huang S, Xu J, Xue Q, Xu T, Shi D, et al. BRG1 improves reprogramming efficiency by enhancing glycolytic metabolism. Cell Mol LIFE Sci. 2024;81(1):482.

2 Zou W, Ding F, Niu C, Fu Z, Liu S. Brg1 aggravates airway inflammation in asthma via inhibition of the PI3K/Akt/mTOR pathway. Biochem Biophys Res Commun. 2018;503(4):3212–8.

3 Dong S, Liang S, Cheng Z, Zhang X, Luo L, Li L, et al. ROS/PI3K/Akt and Wnt/β-catenin signalings activate HIF-1α-induced metabolic reprogramming to impart 5-fluorouracil resistance in colorectal cancer. J Exp Clin Cancer Res. 2022;41(1):15.

4 Gong W, Luo C, Peng F, Xiao J, Zeng Y, Yin B, et al. Brahma-related gene-1 promotes tubular senescence and renal fibrosis through Wnt/β-catenin/autophagy axis. Clin Sci (Lond). 2021;135(15):1873–95.

5 Chen Z, Lai X, Li J, Yuan X, Li Y, Zhang X, et al. BRG1 deficiency promotes cardiomyocyte inflammation and apoptosis by activating the cGAS-STING signaling in diabetic cardiomyopathy. Inflammation. 2025;48(1):299–315.

6 Ge M, Yao W, Yuan D, Zhou S, Chen X, Zhang Y, et al. Brg1-mediated Nrf2/HO-1 pathway activation alleviates hepatic ischemia-reperfusion injury. Cell Death Dis. 2017;8(6):e2841.

7 Sun W, Yu J, Kang Q. Upregulation of heme oxygenase-1 by Brahma-related gene 1 through Nrf2 signaling confers protective effect against high glucose-induced oxidative damage of retinal ganglion cells. Eur J Pharmacol. 2020;875:173038.

**Supplementary Figure S1. Immunofluorescence images showing changes in BRG1 fluorescence intensity following subconjunctival injection.** Groups: control (Ctrl), diabetic (DM), DM + subconjunctival empty vector control (DM+VC), subconjunctival pCMV-Smarca4-3flag-mcmv-EGFP (BRG1-OE), subconjunctival m-Smarca4 shRNA1 (BRG1-KD). Scale bars: 50 μm. BRG1, Brahma-related gene 1; DAPI, 4',6-diamidino-2-phenylindole

**
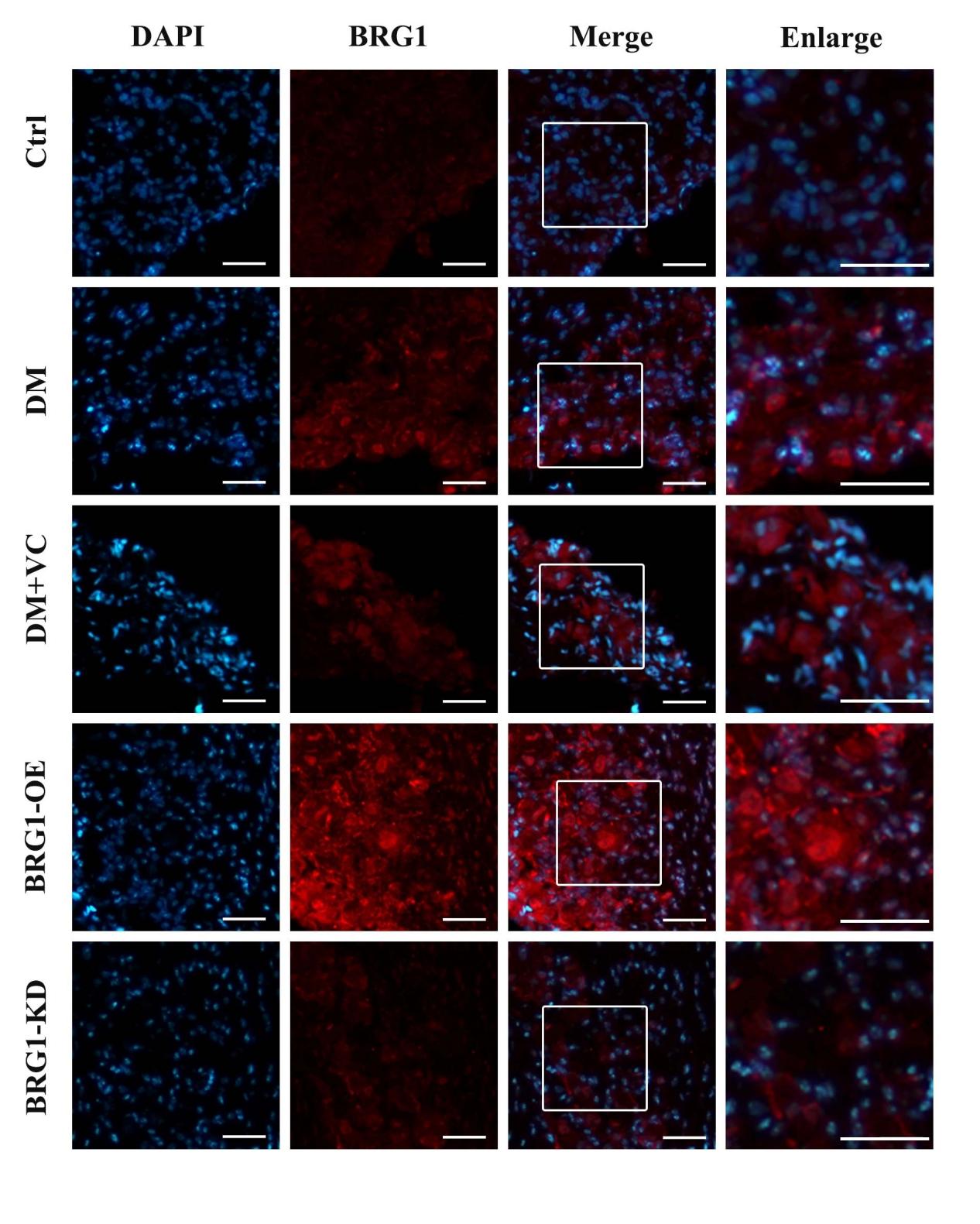
**

**Supplementary Figure S2. Changes in epithelial healing and corneal nerve density after subconjunctival BRG1 intervention. a** Dynamic corneal epithelial healing post-debridement. Groups: control (Ctrl), diabetic (DM), DM + subconjunctival empty vector control (DM+VC), subconjunctival pCMV-Smarca4-3flag-mcmv-EGFP (BRG1-OE), subconjunctival m-Smarca4 shRNA1 (BRG1-KD). **a1** Representative sodium fluorescein staining images at 0, 12, 24, and 36 hours. **a2** Quantified epithelial defect area (%). **b** Corneal nerve regeneration analysis (n = 6). **b1** Representative βIII-tubulin-stained whole-mount images. **b2** Quantitative analysis of corneal nerve fiber density. **c** Corneal sensitivity threshold across groups (n = 6). Scale bars: 50 μm. ns, not significant; *, *P* < 0.05; **, *P* < 0.01; ***, *P* < 0.001; ****, *P* < 0.0001.

**
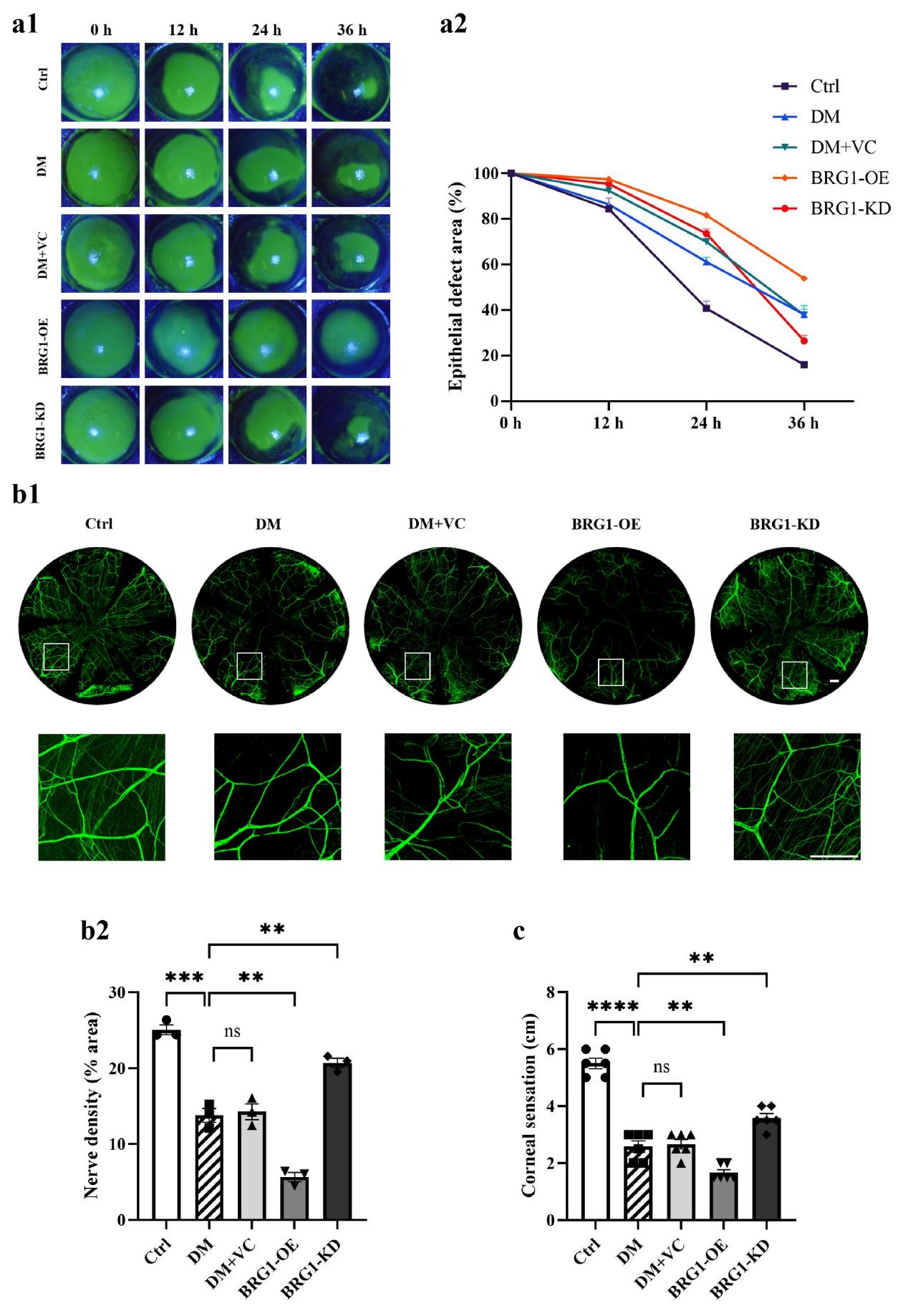
**
